# Hexamethylenediamine-Mediated Polydopamine Film Deposition: Inhibition by Resorcinol as a Strategy for Mapping Quinone Targeting Mechanisms

**DOI:** 10.3389/fchem.2019.00407

**Published:** 2019-06-05

**Authors:** Maria Laura Alfieri, Lucia Panzella, Stefano Luigi Oscurato, Marcella Salvatore, Roberto Avolio, Maria Emanuela Errico, Pasqualino Maddalena, Alessandra Napolitano, Vincent Ball, Marco d'Ischia

**Affiliations:** ^1^Department of Chemical Sciences, University of Naples Federico II, Naples, Italy; ^2^Department of Physics “Ettore Pancini, ” University of Naples Federico II, Naples, Italy; ^3^Institute for Polymers, Composites, and Biomaterials, National Council of Research of Italy (IPCB-CNR), Pozzuoli, Italy; ^4^Faculté de Chirurgie Dentaire, Université de Strasbourg, Strasbourg, France; ^5^Institut National de la Santé et de la Recherche Médicale, Strasbourg, France

**Keywords:** polydopamine, 5, 6-dihydroxyindole, hexamethylenediamine, coating, resorcinol

## Abstract

Hexamethylenediamine (HMDA) and other long chain aliphatic diamines can induce substrate-independent polymer film deposition from dopamine and several other catechols substrates at relatively low concentrations, however the mechanism of the diamine-promoted effect has remained little understood. Herein, we report data indicating that: (a) film deposition from 1 mM HMDA and dopamine is not affected by chemical oxidation with periodate but is markedly inhibited by resorcinol, which also prevents PDA film formation at 10 mM monomer concentration in the absence of HMDA; (b) N-acetylation of HMDA completely inhibits the effect on PDA film formation; (c) HMDA enables surface functionalization with 1 mM 5,6-dihydroxyindole (DHI) polymerization at pH 9.0 in a resorcinol-inhibitable manner. Structural investigation of the polymers produced from dopamine and DHI in the presence of HMDA using solid state ^13^C and ^15^N NMR and MALDI-MS suggested formation of covalent cross linked structures. It is concluded that HMDA enhances polydopamine adhesion by acting both on dopamine quinone and downstream, e.g., via covalent coupling with DHI. These results provide new insights into the mechanisms of PDA adhesion and disclose resorcinol as a new potent tool for targeting/mapping quinone intermediates and for controlling polymer growth.

## Introduction

The substrate and material-independent adhesion and deposition of catechol-based thin films and coatings under wet conditions is a topic of growing interest in materials science for a broad range of technological and biomedical applications (Suárez-García et al., 2017; d'Ischia and Ruiz-Molina, 2018). Paradigms of catechol-based adhesion are found in mussel foot proteins, which account for byssus sticking to rocks, and which inspired the development of polydopamine (PDA), a black insoluble eumelanin-like material (Ito et al., 2011) with strong substrate-independent underwater adhesion and film-forming properties.

**Scheme 1:**
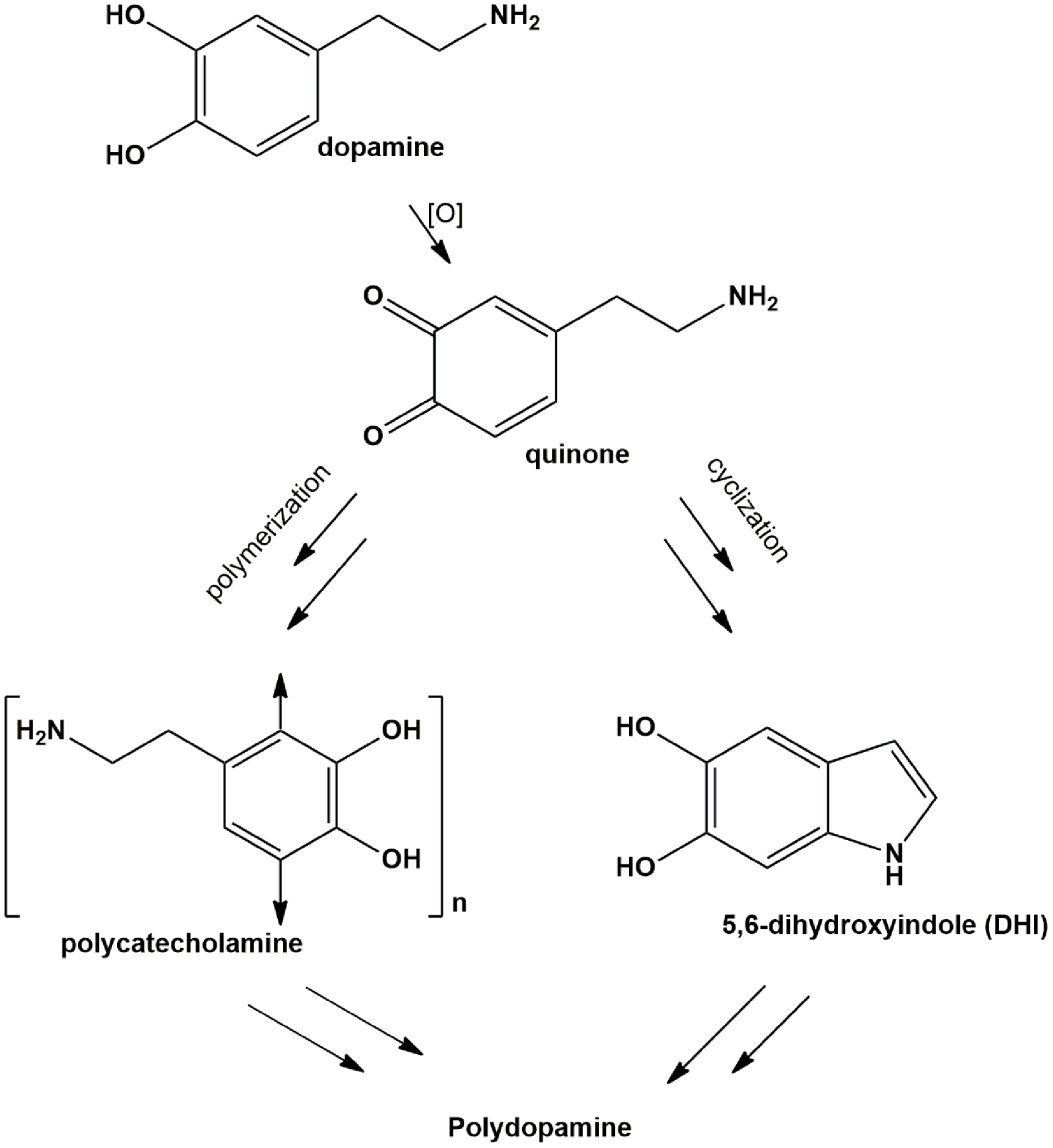
Synthetic pathways for polydopamine formation.

Over the past few years, advances into the mechanisms underlying PDA adhesion properties pointed to a complex interplay of structural factors and mechanisms that remain, however, still obscure (Lee et al., 2011; Ball et al., 2012; Ryu et al., 2018; Salomäki et al., 2018). In a recent study (Alfieri et al., 2018a,b), it was shown that (a) PDA coating formation at pH 9.0 in carbonate buffer requires dopamine concentrations >1 mM; (b) is due to species produced in the early stages of dopamine autoxidation but is not attributable to cyclized 5,6-dihydroxyindole (DHI) intermediates produced by dopamine autoxidation, as DHI melanin does not display appreciable adhesion; (c) is accelerated by periodate at pH 9.0 causing fast conversion to the *o*-quinone (Scheme 1). A most relevant finding was that hexamethylenediamine (HMDA) and other long chain aliphatic (di)amines enable deposition of PDA films under low dopamine concentration conditions (<1 mM) where no UV detectable coating is normally observed.

This observation was of considerable interest for two main reasons: (a) it suggested that long chain flexible cross-linking systems play an important role in adhesion mechanisms evidently compensating the drop in bimolecular coupling processes at low dopamine concentrations; (b) proper selection of diamine additives can substantially widen the scope of PDA-based surface functionalization technologies and can pave the way for more versatile coating methodologies based on mixtures of cross-linking and functionalizing additives.

The role of diamines as adhesion enhancers for catechol-based polymers is well-documented. Chen et al. (2014) first reported that copolymerization of gallic acid (GA) and HMDA leads to films that can be deposited on various surfaces. Addition of HMDA during PDA deposition was also proposed as a means of obtaining films rich in amine groups, with a high cross-linking degree and resistance to hydrolysis and swelling (Yang et al., 2015).

Chemically stable functional biocompatible thin films were obtained by autoxidation of 1 mM caffeic acid at pH 9.0 in the presence of equimolar amounts of HMDA (Iacomino et al., 2017). Finally, functionalizable coatings were reported by a cross-linking reaction between pyrocatechol and HDMA under oxidizing conditions (Suárez-García et al., 2017).

**Scheme 2:**
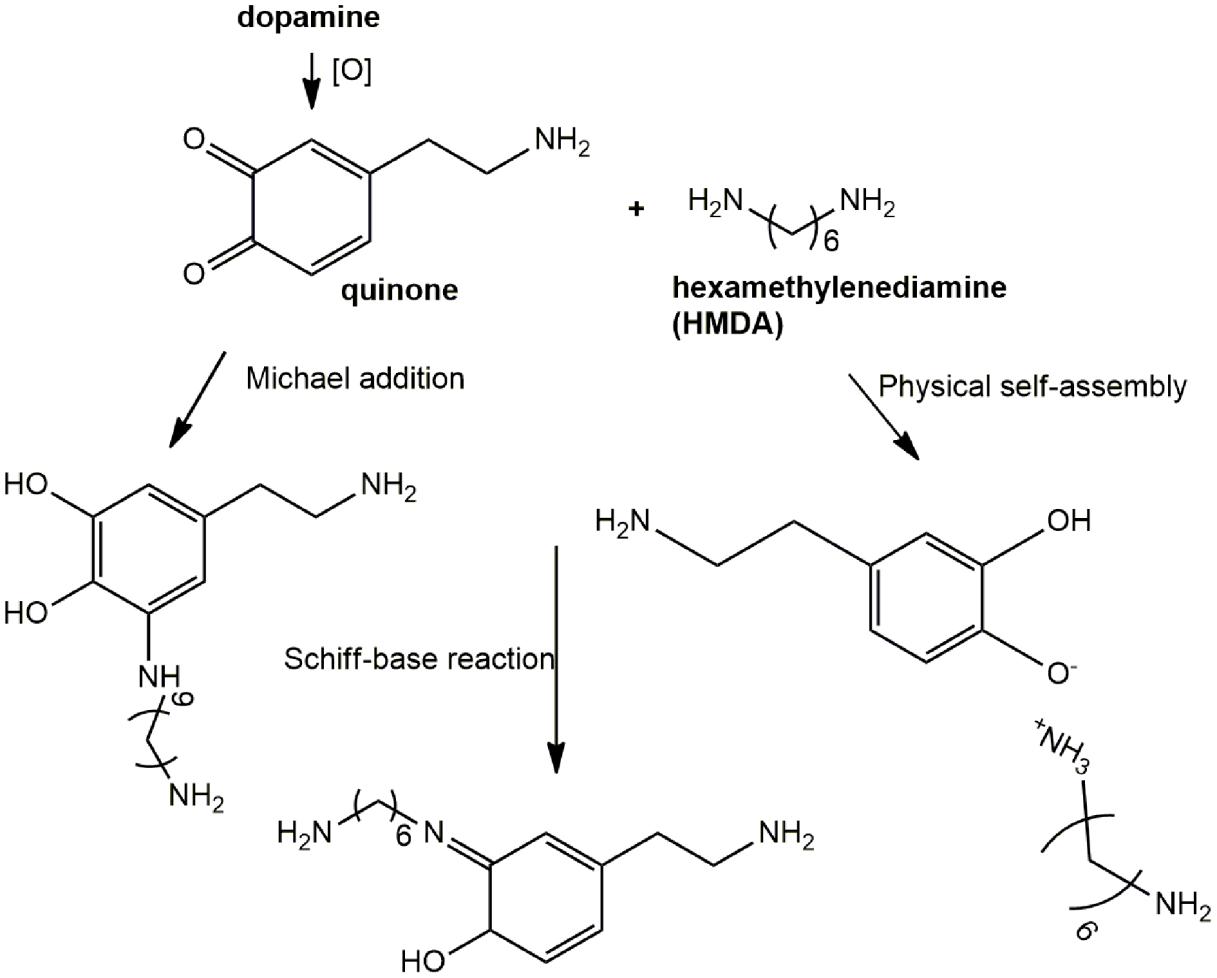
Schematic outline of possible mechanisms underlying HMDA-promoted film deposition from dopamine.

In all these studies, it was generally proposed that HMDA gives rise to intermolecular amine-quinone condensation processes leading to highly cross-linked oligomer structures. However, the detailed mechanism by which HMDA is able to mediate PDA deposition under low concentration conditions, e.g., 1 mM, has remained so far unknown. Deposition of PDA at low monomer concentration would be beneficial due to the lower mass waste associated with bulk polymer precipitation and considering the notorious toxicity of dopamine. An interesting, most relevant issue, concerns the nature of the main quinone target(s) of HMDA along the PDA formation pathway. The previously reported MALDI-TOF analysis of PDA-HMDA films in Tris buffer suggested that HMDA is incorporated into PDA via covalent bondings to both cyclized and uncyclized quinone moieties, e.g., by Michael addition and Schiff-base reactions, as evidenced by peaks at 268, 252, and 374 m/z, and physical self-assembly, by electrostatic interactions of −NH${}_{3}^{+}$ (Yang et al., 2015) (Scheme 2). A significant incorporation of Tris buffer into the PDA films was also apparent (Della Vecchia et al., 2014), due to a competition with HMDA.

A relevant mechanistic point for discussion, in this regard, concerns the possible competition between intermolecular amine-quinone conjugation *vs*. intramolecular cyclization of dopamine quinone to give aminochrome and indole-type intermediates.

Alternatively, HMDA can attack cyclized quinone-containing indole units, e.g., aminochrome and 5,6-indolequinone, two possible targets downstream in the pathway, but direct experimental evidence supporting this view is so far missing. The main goal of the present paper is to investigate the mechanisms of HMDA-mediated PDA deposition as a means of gaining deeper insights into the structural factors underlying PDA adhesion. Specific aims of the study include: (1) an assessment of the inhibitory effects the quinone trapping agent resorcinol (Crescenzi et al., 1991; Iacomino et al., 2019) as a tool to probe the role of quinone intermediates in PDA film formation and in HMDA-mediated coating at low dopamine concentration; (2) the investigation of the ability of HMDA to induce film formation from DHI, which has been shown to give eumelanin-type polymers devoid of adhesion properties under PDA deposition conditions (Alfieri et al., 2018a).

## Experimental

All reagents were purchased from commercial sources (Sigma-Aldrich and AnalytiCals Carlo Erba) and used without further purification. Ultraviolet-visible (UV-vis) spectra were run on a V-730 Jasco instrument. Quartz substrates were cleaned by soaking in piranha solution (96% H_2_SO_4_/30% H_2_O_2_ 5:1 v/v) overnight, rinsed with distilled water and dried under vacuum.

### Synthesis of PDA and General Procedure for Coating Experiments

Polydopamine was prepared as previously reported (Lee et al., 2007) by autoxidation of dopamine hydrochloride (100 mg, 0.5 mmol) in 0.05 M carbonate buffer (pH 9.0) (final concentration 1 mM or 10 mM), under vigorous stirring. Quartz substrates were dipped in the autoxidation mixtures after complete dissolution of the catechol. After 24 h, substrates were rinsed with distilled water, sonicated in methanol/water solution 1:1 v/v, and air-dried. The coated substrates thus obtained were analyzed by UV-vis spectrometry. When required, after 24 h the reaction mixture was acidified to pH = 2.0 with 4 M HCl, centrifuged at 7,000 rpm at 4°C for 15 min and the precipitate washed three times with water and lyophilized to collect the dark pigment (45% w/w yield).

### HMDA-Promoted Film Formation From Dopamine or DHI

To a 1 mM solution of HMDA, dopamine (200 mg) or DHI (200 mg), prepared according to Edge et al. (2006), were added under vigorous stirring in a 1:1 molar ratio. Quartz substrates were dipped into the reaction mixture and left under stirring for the appropriate time interval, then rinsed with distilled water, sonicated, dried, and analyzed as above.

In another set of experiments, to 1 mM dopamine solution (50 mg, 0.26 mmol) or DHI solution (50 mg, 0.34 mmol) containing 1 mM HMDA (30 mg) in 0.05 M carbonate buffer pH = 9.0, resorcinol (1 or 5 mM), as an inhibitor agent, was added and the mixture was left under stirring for 24 h. Control reactions with 10 mM dopamine were run in the presence and in the absence of resorcinol. Quartz substrates were dipped into the reaction mixture and left under stirring for 24 h.

In other experiments, 30 mg of HMDA were acetylated with 500 μL of acetic anhydride and 50 μL of pyridine at room temperature overnight. The product thus obtained was added to a 1 mM dopamine solution in 0.05 M carbonate buffer, pH = 9.0. A quartz substrate was dipped in the reaction mixture and left under stirring for 24 h and then analyzed as above.

In other experiments PDA synthesis and deposition was investigated by adding periodate to an equimolar (1 mM) mixture of HMDA and dopamine at pH 9.0 in carbonate buffer or at pH 5.0 in acetate buffer.

### Sample Preparation for MALDI-MS Analysis

One milliliter of the analyte (PDA or DHI melanin solubilized in dimethylsulfoxide and homogenized with a glass/glass potter) was premixed with 1 mL of the matrix (2,5-dihydroxybenzoic dissolved in water), and then 2 μL of the resulting mixture were pipetted on the MALDI target plate and air-dried for MALDI-ToF MS analysis. MALDI spectra were recorded on a Sciex 4800 MALDI ToF/ToF instrument. The laser was operated at 3,700 Hz in the positive reflectron mode. The mass spectrometer parameters were set as recommended by the manufacturer and adjusted for optimal acquisition performance. The laser spot size was set at medium focus (B50 mm laser spot diameter). The mass spectra data were acquired over a mass range of *m*/*z* 100–4,000, and each mass spectrum was collected from the accumulation of 1,000 laser shots. Raw data were analyzed using the computer software provided by the manufacturer and reported as monoisotopic masses.

### Atomic Force Microscopy and Micro-Raman Analysis

The combined atomic force microscopy (AFM) and micro-Raman analysis were conducted with the integrated apparatus Alpha300 RS. The system can be switched at will between AFM and confocal micro-Raman configurations, allowing a combined topographical and spectral characterization of a specified microregion of the sample. The samples topographies were studied by AFM in intermittent contact (AC) mode using a cantilever with 75 kHz resonant frequency. For the micro-Raman analysis, a laser beam at λ = 488 nm was used as excitation light source. The beam was focused onto the sample surface by means of a 50 × microscope objective (numerical aperture of 0.75) working in epi-illumination mode. The diffraction-limited focused spot in the objective focal plane had a full width at half-maximum (FWHM) of approximately 320 nm. The light backscattered from the sample was collected by the same objective, and sent to the spectrograph through a confocal optical collection path. The samples analysis was conducted in the microregions marked by the colored squares in the optical images of the films. The AFM images correspond to an area of 25 × 25 μm^2^ (yellow square). For the micro-Raman imaging, the samples were scanned over the area of 18 × 18 μm^2^ indicated by the red squares. The scanning step was set at 320 nm in order to match the FWHM of the diffraction-limited focused laser spot. The Raman spectra results from 1 s acquisition time, while the Raman images of the analyzed regions were reconstructed integrating for each scanned position the Raman signal in a spectral window of 110 cm^−1^ in width, centered at the 1,590 cm^−1^ peak.

### Solid State Nuclear Magnetic Resonance

Solid state nuclear magnetic resonance (NMR) spectra were recorded on a Bruker Avance II 400 spectrometer equipped with a 4 mm MAS probe. Samples were packed into 4 mm zirconia rotors sealed with Kel-F caps and the spinning speed was set at 10 and 6 kHz for ^13^C and ^15^N NMR experiments, respectively. Cross polarization (CP) spectra were recorded with a variable spin-lock sequence and a relaxation delay of 4s; a ^1^H π/2 pulse width of 3.8 μs was employed and high- power proton decoupling was applied during acquisition. For ^13^C spectra, the contact time was set to 2 ms and 20,000 scans were recorded per each sample. Spectra were referenced to external adamantane (CH_2_ signal 38.48 ppm downfield of tetramethylsilane (TMS), set at 0.0 ppm). For ^15^N spectra, the contact time was set to 1.5 ms and 60,000 scans were recorded. Spectra were referenced to external glycine (amine signal 32.6 ppm downfield of ammonia, set at 0.0 ppm).

## Results and Discussion

### Effect of Diamine Modification and Experimental Conditions on HMDA-Mediated PDA Film Deposition

In an initial set of experiments the effects of structural modifications and experimental protocol variations on HMDA-mediated PDA film deposition were investigated under the selected conditions of 1 mM dopamine concentration in carbonate buffer at pH 9.0. To assess the role of free amine groups, the diacetyl derivative of HMDA was prepared and allowed to react at equimolar concentration with 1 mM dopamine at pH 9.0. No detectable PDA deposition was observed, confirming the importance of the free amine groups for HMDA-mediated adhesion (for details see Figure S1).

**Figure 1:**
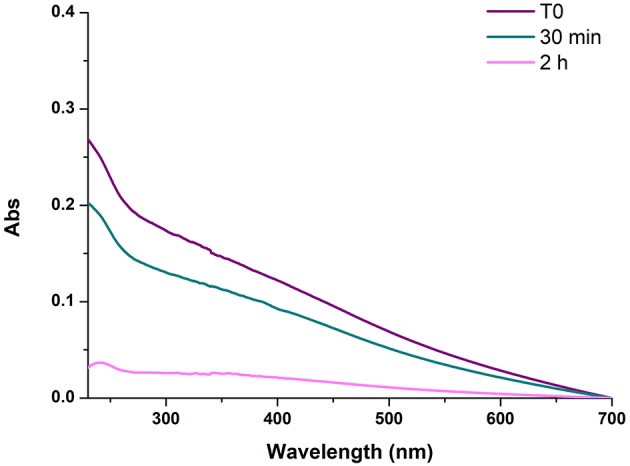
UV-vis spectra of PDA/HMDA films with diamine added at different times after the beginning of dopamine autoxidation. Each curve refers to a separate experiment in which HMDA was added at the stated time. T0 refers to the reference experiment in which HMDA was added at the beginning of the reaction.

On this basis, further experiments were aimed at determining the temporal dependence of the diamine-mediated effects, a parameter that is related to the evolution of reaction intermediates with the progress of oxidation. To determine the degree of PDA film deposition the absorption profile of the quartz substrates was taken as an approximate but convenient index of film thickness since, as discussed in previous studies, substrate absorption data were found to satisfactorily correlate with AFM analyses (Alfieri et al., 2018b). Under dip coating conditions, PDA-coated quartz surfaces displayed virtually featureless absorption profiles akin to those of PDA suspensions. Comparative analysis of film deposition was conveniently carried out by taking absorbance values at 400 nm where no monomer or additive can affect PDA absorption. Addition of 1 mM HMDA 30 min after the autoxidation of 1 mM dopamine at pH 9.0 had started resulted in films displaying lower absorbance at 24 h compared to the standard conditions (T = 0). A much more pronounced decrease in film absorbance was determined when HMDA was added after 2 h, supporting the view that the main target species for HMDA amine groups are generated in the early stages of the autoxidation process and that, when the oxidation proceeds further, precipitation of the polymer makes HMDA ineffective in inducing adhesion (Figure 1).

### The Role of Quinones in HMDA-Mediated PDA Film Formation: Fast Generation by Periodate Oxidation at Acidic and Alkaline pH

**Figure 2:**
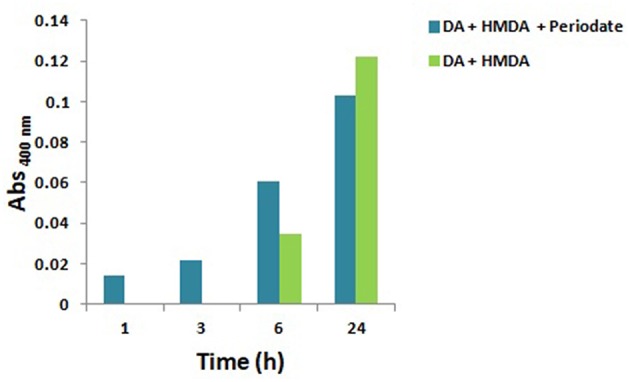
Kinetics of PDA/HMDA film formation: absorbance of the films obtained in the absence or in the presence of periodate in 0.05 M carbonate buffer pH 9.0 at a selected wavelength (400 nm). No detectable absorption was measured in the autoxidation experiments in the absence of HMDA.

Since quinones are the most reasonable targets for HMDA in film deposition at low dopamine concentration, the effect of fast and efficient quinone formation on film deposition was investigated by oxidizing an equimolar (1 mM) mixture of HMDA and dopamine at pH 9.0 in carbonate buffer with 1 mM sodium periodate. A marked acceleration of the deposition kinetics was observed with respect to the auto-oxidative process in the first 3 h (Figure 2). These results confirmed the role of HMDA as enhancer of film deposition also under oxidation conditions leading to fast quinone generation.

To assess whether HMDA can exert its role also at acidic pH, under conditions of substantial amine protonation, PDA deposition experiments were performed by periodate-induced oxidation of 1 mM dopamine in acetate buffer at pH 5.0, under previously reported conditions (Ponzio et al., 2016), in the presence of HMDA.

The results reported in Figure S2 indicated that film deposition is observed only after 3 h, in contrast to what observed at alkaline pH. The slower kinetics of film deposition determined under acidic conditions can be explained by extensive protonation of HMDA hindering chemical coupling with dopamine quinone and other quinone intermediates in the PDA deposition pathway. By a similar effect, intramolecular cyclization and intermolecular crosslinking of dopamine quinone units may be slowed down with a consequent inhibitory effect on the coating process (Alfieri et al., 2018b).

### The Role of Quinones in HMDA-Mediated PDA Film Formation: Effect of Resorcinol as Dopamine Quinone Trapping Agent

In other experiments the effects of resorcinol, a well-known dopamine quinone trapping agent (Crescenzi et al., 1991; Acuña et al., 2013; Iacomino et al., 2019), on deposition of PDA films from 1 mM dopamine in the presence of HMDA was investigated.

**Figure 3:**
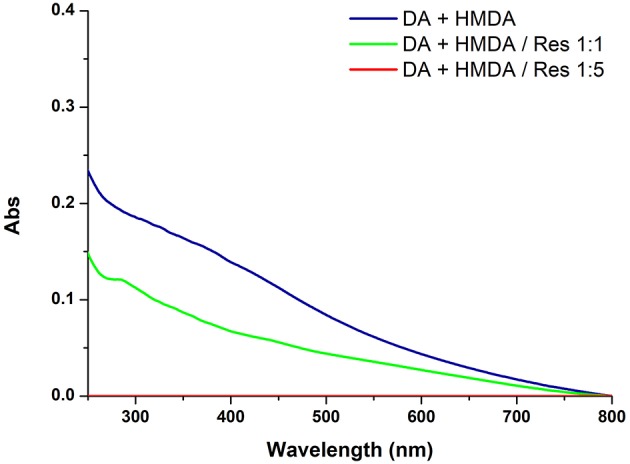
UV-vis spectra of quartz substrates dipped for 24 h into 1 mM solution of DA with 1 mM HMDA in the absence or in the presence of 1 or 5 mM resorcinol (Res).

Figure 3 shows the effects of 5 and 1 mM resorcinol on film deposition from 1 mM dopamine and 1 mM HMDA.

The results revealed a marked concentration dependent inhibition of film deposition by resorcinol. Assuming a rough proportionality between film absorbance and thickness, it can be concluded that resorcinol competes with HMDA in the reaction process preventing the generation of adhesive species and blocking the growth of the film via addition to dopamine quinone. In support of this conclusion, spectrophotometric analysis of the supernatant from the reaction mixture (for details see Figure S3) indicated a yellow chromophoric species with an intense emission at 460 nm compatible with the azamonardine-type adduct featuring a methanobenzofuroazocinone scaffold that has been described to arise from trapping of dopaminoquinone by resorcinol (Scheme S1) (Acuña et al., 2013).

**Figure 4:**
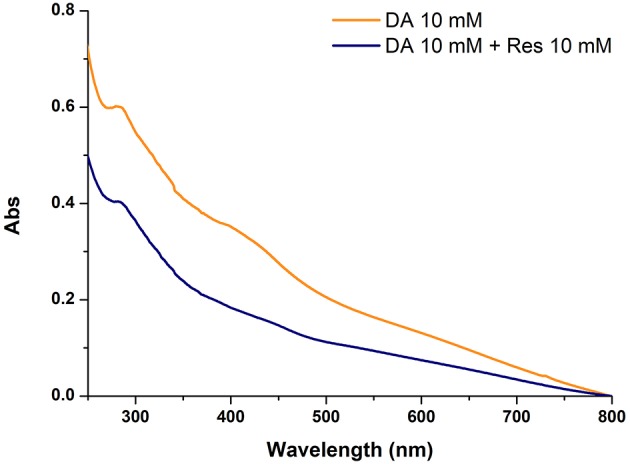
Uv-vis absorption spectra of quartz substrates subjected to dip-coating for 24 h with 10 mM dopamine in the absence and in the presence of equimolar amounts of resorcinol (Res).

To confirm the inhibitory role, in other experiments the effect of resorcinol on PDA film adhesion was measured under the usual dopamine concentration of 10 mM (Lee et al., 2007) with equimolar inhibitor and in the absence of HMDA. A marked, though not complete, inhibition of film deposition was again observed, suggesting coupling of resorcinol with quinone intermediates involved in adhesion and cross-linking (Figure 4).

Overall these results provide evidence that resorcinol can target dopamine quinone (as apparent from the azamonardine fluorophore formation) hindering adhesion-producing coupling with HMDA, but do not allow to settle a critical issue relating to the actual role of dopamine quinone in the adhesion process, i.e.,: is the quinone *the main structural determinant* of adhesion, being directly involved in coupling with amine-containing chains, or is it *the precursor* of the species serving as the actual target for the amine groups involved in adhesion and cohesion?

The inhibitory effect of resorcinol on HMDA-induced film deposition with 1 mM dopamine reflects the competition between HMDA and resorcinol for nucleophilic attack, but the concentration-dependence of the effect points either to a partial escape of dopamine quinone from trapping by resorcinol and/or to an adhesion-promoting effect of the diamine also downstream to dopamine quinone. In that case, an excess of resorcinol would be necessary to completely prevent HMDA conjugation *at all stages* of the dopamine polymerization process, i.e., by coupling with all other quinones produced after the dopamine quinone stage.

Indeed, the partial inhibition of PDA deposition at 10 mM concentration by equimolar resorcinol, i.e., under conditions where efficient trapping of dopamine quinone is usually observed, would provide support to a more complex effect of resorcinol than the simple trapping of dopamine quinone. Among possible targets produced downstream dopamine quinone generation, the aminochrome was at first considered to be of lesser relevance, since aminochromes display, despite a complex chemistry (d'Ischia et al., 1988) a lesser electrophilic character than o-quinones by virtue of their 4-aminoquinone character (this issue is the focus of ongoing separate studies). Conversely, the quinone from DHI was considered a likely target of HMDA. Accordingly, the effect of HMDA on film deposition by autoxidation of DHI was next investigated since it is a species unable *per se* to produce coating deposition and is a chief precursor of eumelanin polymers (Ito et al., 2011). Like other eumelanin precursors, DHI is endowed with biologically and technologically relevant properties, including antioxidant effects (Memolia et al., 1997), reactivity with biomolecules (Bisaglia et al., 2007), and susceptibility to solid state polymerization for melanin-based films and coating (Pezzella et al., 2015).

#### Effect of HMDA on Film Deposition From DHI at pH 9.0

**Figure 5:**
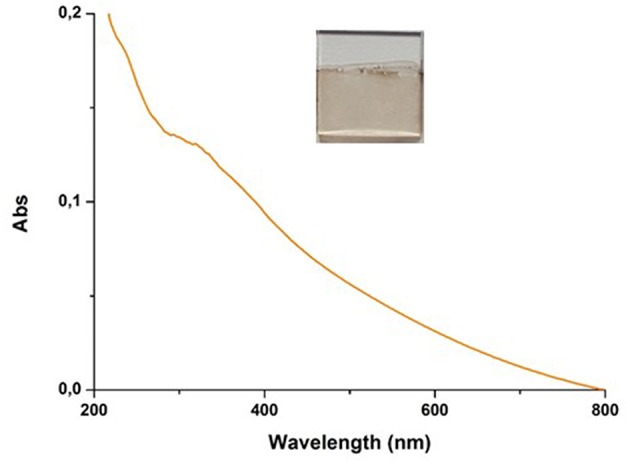
UV–vis spectra of quartz substrate dipped into 1 mM solution of DHI in 0.05 M carbonate buffer (pH 9.0) in the presence of equimolar amount of HMDA over 24 h of DHI/HMDA coated coverslip.

As previously observed, autoxidation of 1 mM DHI in carbonate buffer did not result in UV-detectable film deposition. However, addition of 1 mM HMDA led to the deposition of a dark coating resembling PDA (Figure 5).

**Figure 6:**
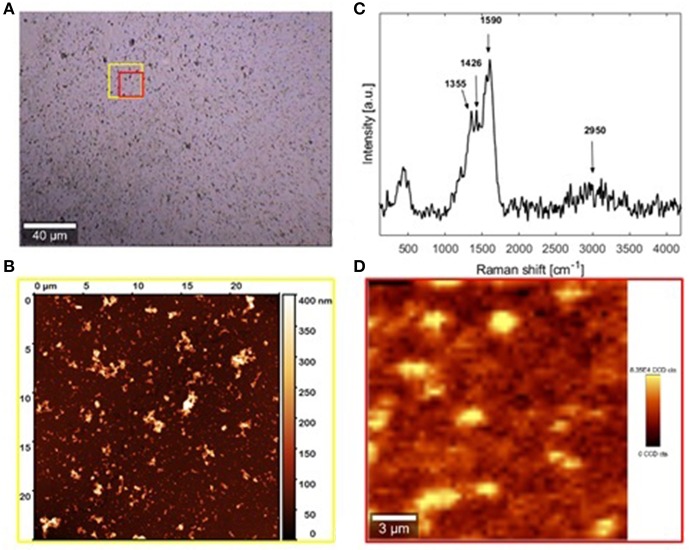
AFM and micro-Raman analysis of the sample DHI/HMDA **(A)** Bright-field image of the investigated sample region collected by 20× microscope objective. **(B)** Raman spectrum **(C)** AFM image of the area indicated by the yellow square in the optical image. **(D)** Micro-Raman image relative to the red sample region in the optical image. *FILM THICKNESS: 30* ±*10 nm*.

DHI/HMDA melanin films were then characterized by AFM coupled with micro Raman analysis (Figure 6). The Raman spectrum resulting from the average of 500 spectra collected in different positions of the sample is shown in Figure 6C, together with the positions of the observable peaks. Main bands at 1,355, 1,426, and 1590 cm^−1^ were detected, which were compatible with the presence of aromatic rings, while the broadband around 2,900 cm^−1^ was attributable to strongly hydrogen-bonded OH and NH stretching vibrations. Notably, no intense carbonyl band in the 1,650–1,700 cm^−1^ range was detected. As apparent from the optical and AFM images (Figures 6A,B, respectively), a uniform film with good grain distribution and dispersion was achieved. The main estimated thickness was 30 ± 10 nm. The origin of the large roughness of the DHI/HMDA films could be explained by considering both the low solubility of DHI and its oligomers and its fast rate of oxidation leading to fast deposition of aggregates on the growing films.

Addition of equimolar amounts of resorcinol to the DHI solution in the presence of HMDA completely inhibited DHI melanin deposition on the quartz substrates despite significant polymer precipitation. Considering the ability of resorcinol to completely block film deposition from DHI but not from dopamine, it can be argued that in the case of dopamine part of the quinone escapes coupling with resorcinol and can still undergo (a) coupling with HMDA, (b) intermolecular cross-linking, and (c) intramolecular cyclization to give aminochrome and then DHI. On this basis, the present results suggest that resorcinol is capable of bringing nucleophilic attack onto indolequinone intermediates, and that this attack is more efficient than at the dopamine quinone level. This view is consistent with the notorious instability of 5,6-indolequinone which cannot be isolated or even identified under standard reaction conditions. Thus, it can be suggested that DHI/HMDA coatings were made by conjugates of HMDA with DHI and oligomers thereof.

#### Structural Characterization and Properties of PDA and DHI Melanins Produced in the Presence of HMDA

**Figure 7:**
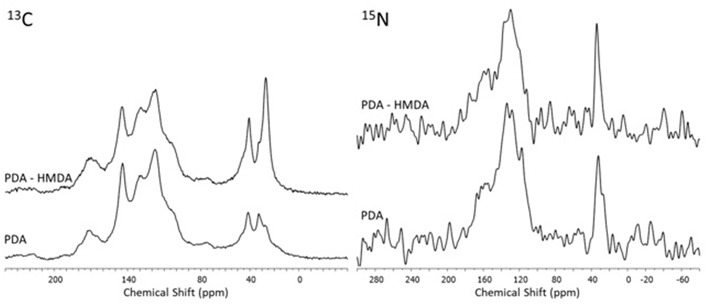
^13^C and ^15^N NMR spectra of PDA obtained in presence and in absence of the diamine (HMDA).

**Figure 8:**
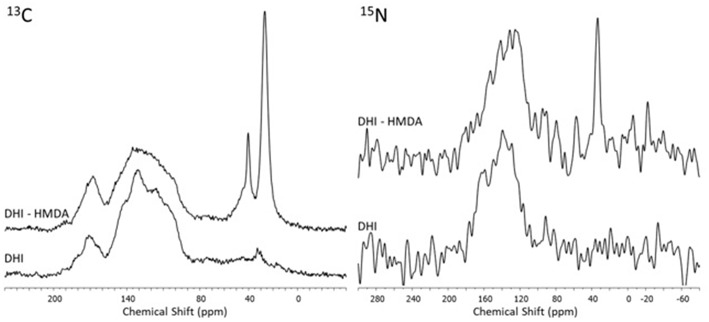
^13^C and ^15^N NMR spectra of the oxidation products of DHI obtained in presence and in absence of the diamine (HMDA).

Insight into the structure of the PDA and DHI melanins samples produced in the presence and in the absence of HMDA was then obtained by solid state ^13^C and ^15^N NMR analysis of the bulk materials that precipitated from the reaction mixtures (Figures 7, 8). The ^13^C NMR spectrum of neat PDA is consistent with the products of dopamine autoxidation as observed in previous studies (Della Vecchia et al., 2013). The PDA sample obtained in the presence of HMDA showed a change in intensity and position of the main aliphatic resonances, centered at around δ = 27, 32, and 41 ppm, while the aromatic region presents an essentially unchanged pattern of signals. The ^15^N NMR spectrum consistently indicated a modest enhancement in the aliphatic amine component relative to the aromatic component in the presence of HMDA. More pronounced HMDA-dependent differences were observed in the case of DHI melanin, for which distinct aliphatic resonances were appeared in both the ^13^C and ^15^N NMR spectra only in presence of HMDA. An increase in the relative intensity of carbonyl/carboxyl signal was also observed in the amine-containing sample. On a comparative basis, the most remarkable finding was that, within the inherent limitations of peak area integration data for solid state ^13^C and ^15^N NMR spectra, the apparent degree of incorporation of HMDA into the polymer bulk structure was lower in the case of PDA compared to DHI melanin. This observation, which is especially supported by ^15^N NMR analysis, would suggest that HMDA incorporation is not so important at the dopamine quinone level as it could be at the later indolequinone stage. It is worth mentioning that the covalent coupling of nucleophilic agents with 5,6-indolequinone is well-documented in the literature (Napolitano et al., 1996). It should be considered, however, that these data refer to the *bulk polymer*, which may be different from the adhesive film, so further work is required to definitively assess the degree of incorporation of HMDA at the various stages of dopamine oxidation and its actual relevance to adhesion processes.

**Figure 9:**
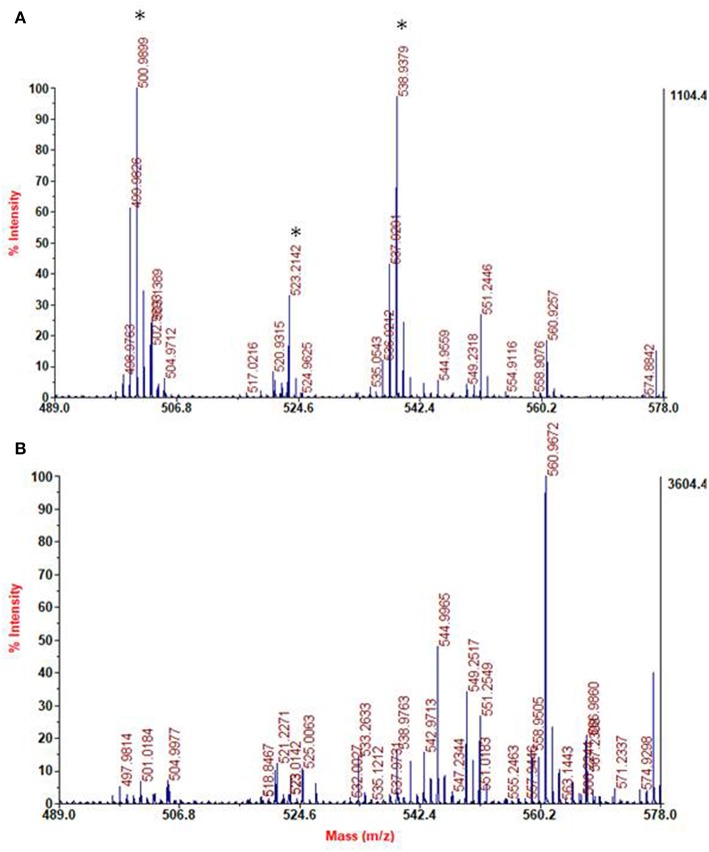
Segmental spectra of MALDI-MS spectra of the solid separated from PDA/HMDA **(A)** and PDA **(B)** mixture after centrifugation. Asterisks indicate specific peaks of PDA/HMDA.

**Figure 10:**
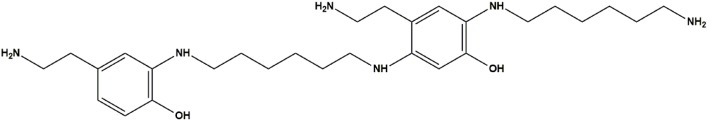
Tentative structure representative of the possible species responsible for the peak at *m*/*z* 501 in the PDA/HMDA mixture.

The bulk precipitates of the 1 mM dopamine oxidation mixture in the presence and in the absence of HMDA were also subjected to MS analysis in the MALDI-ToF mode after centrifugation and extensive washings, using 2,5-dihydroxybenzoic acid as the matrix. PDA samples were applied on the target plate from fine suspensions in DMSO. Interestingly the spectrum of the PDA/HMDA melanin (Figure 9A) showed an intense peak at [M+H]^+^ = m/z 501 (+ Na^+^, +K^+^) which was missing in the control PDA sample (Figure 9B). This peak was suggestive of two catecholamine units and two HMDA moieties linked via loss of two oxygen atoms, suggesting dominant condensation of the amine groups with the carbonyl moieties in dopamine quinone rather than addition at conjugated positions. A tentative structure compatible with a pseudomolecular ion peak at *m*/*z* 501 is shown in Figure 10.

**Figure 11:**
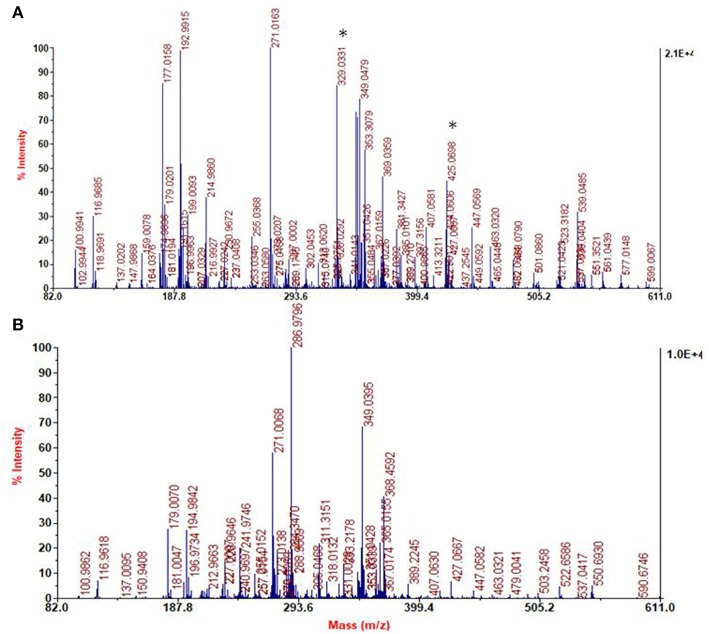
Segmental spectra of MALDI-MS of the solid separated from DHI/HMDA **(A)** and DHI melanin mixture after centrifugation **(B)**. Asterisks indicate specific peaks of DHI/HMDA.

**Figure 12:**
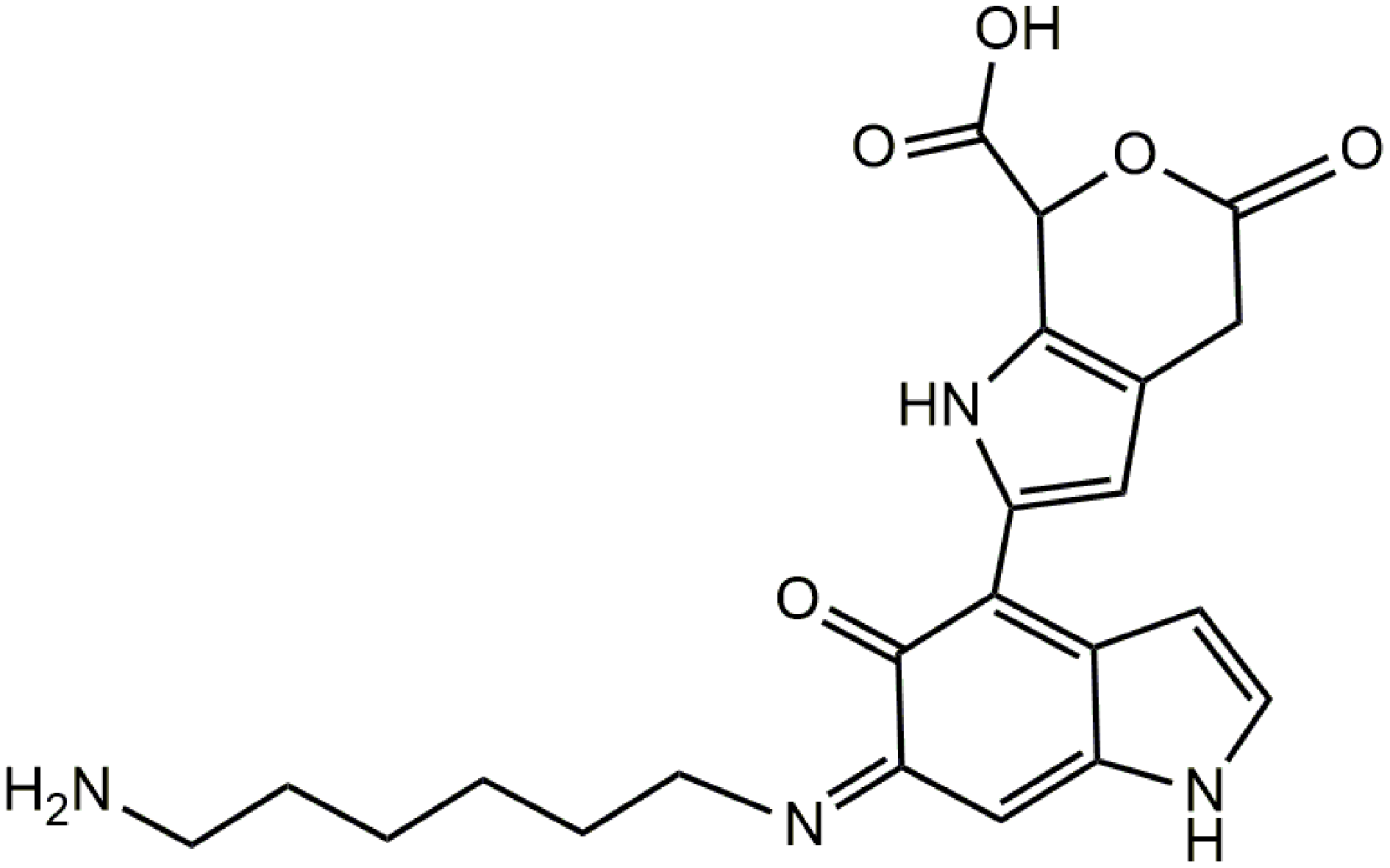
Tentative structure representative of the possible species responsible for the peak at *m/z* 425 in the PDA/HMDA mixture.

The spectra of DHI melanin is mainly characterized by peaks attributable to oxidative degradation. Interestingly the spectrum of the DHI/HMDA melanin (Figure 11A) showed an intense peak at [M+H]^+^ = m/z 425 which was missing in the control DHI sample (Figure 11B). This peak was suggestive of a 5,6-dihydroxyindole unit, a cleaved indolequinone and one HMDA moieties linked via loss of two oxygen atoms, suggesting also in this case a dominant condensation of the amine groups with the carbonyl moieties rather than addition at conjugated positions. In support of this hypothesis, the spectrum shows also an intense peak at [M+H]^+^ = m/z 329 suggestive of the same indole units without the HMDA chain. A tentative structure compatible with a pseudomolecular ion peak at *m*/*z* 425 is shown in Figure 12.

## Conclusions

Although the detailed mechanisms of the adhesion and cohesion phenomena responsible for PDA coating have not yet been clarified, several important advancements can be made by suitable model experiments aimed at identifying the key structural determinants of film deposition under wet conditions. When discussed in the light of available data from previous studies, the results reported in this paper can overall be taken to make a series of relevant conclusions as detailed in the following.

**Scheme 3:**
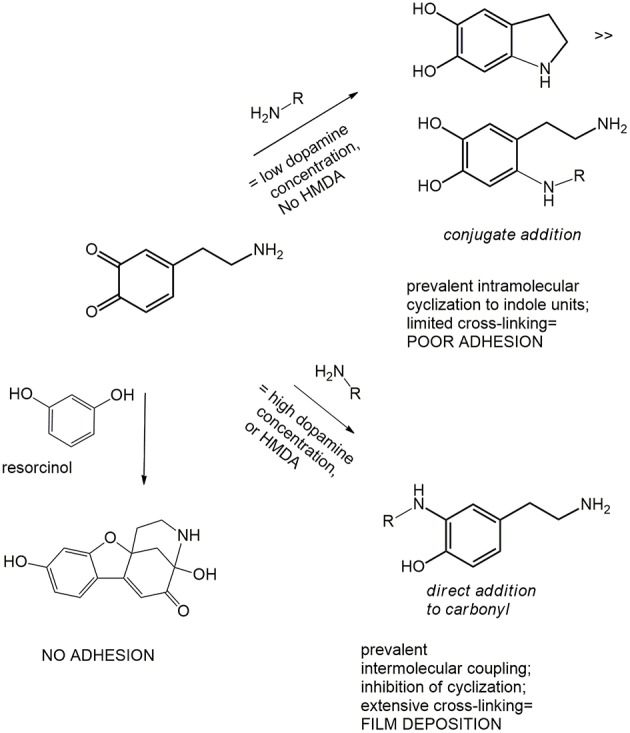
Schematic outline of possible mechanisms underlying film deposition from dopamine or DHI.

- Attack of amine groups onto quinone moieties leading to *o*-quinoneimine intermediates may occur to a significant degree via condensation at carbonyl sites with loss of oxygen in the form of water (MS evidence). This mechanism would explain the concentration dependence of film deposition from dopamine, since conjugated attack onto the carbonyl positions would invariably be favored in an intramolecular rather than intermolecular fashion for entropic reasons, whereby concentration dependence would expectedly be minimal in case of exclusive or dominant conjugate attack (Scheme 3).
- Resorcinol, a dopamine quinone trapping agent, is reported to inhibit PDA film deposition both in the presence and in the absence of HMDA. This effect is a further indirect evidence for the involvement of quinone moieties in film formation (Scheme 3).
- DHI is shown for the first time to form adhesive films under dip coating conditions in the presence of HMDA and in a resorcinol-inhibitable manner. The observation that the inhibitory effect of resorcinol on HMDA-mediated film deposition is much more pronounced in the case of DHI than of dopamine suggests that DHI-derived quinones are at least as important as dopamine quinone as targets for amine-related crosslinks in film deposition (Scheme 3).

Besides corroborating the central role of quinone intermediates in film formation processes, the results of this study highlighted the possible use of resorcinol as a valuable tool to probe the mechanisms of PDA film deposition, and for the functionalization and control of film growth.

## Data Availability

The raw data supporting the conclusions of this manuscript will be made available by the authors, without undue reservation, to any qualified researcher.

## Author Contributions

AN, LP, and MI: conceptualization. MA, LP, MS, SO, RA, and ME: methodology. SO, PM, RA, and ME: data curation. MA, LP, ME, AN, PM, and MI: writing—original draft preparation. VB, MI, and MA: writing—review and editing.

### Conflict of Interest Statement

The authors declare that the research was conducted in the absence of any commercial or financial relationships that could be construed as a potential conflict of interest.
