# Supplementary material for: Hexamethylenediamine-Mediated Polydopamine Film Deposition: Inhibition by Resorcinol as a Strategy for Mapping Quinone Targeting Mechanisms
Source: Front Chem. 2019 Jun 5;7:407. doi: 10.3389/fchem.2019.00407 (PMC6560077; doi:10.3389/fchem.2019.00407)
Supplement: Supplementary file 1 [file Data_Sheet_1.docx]

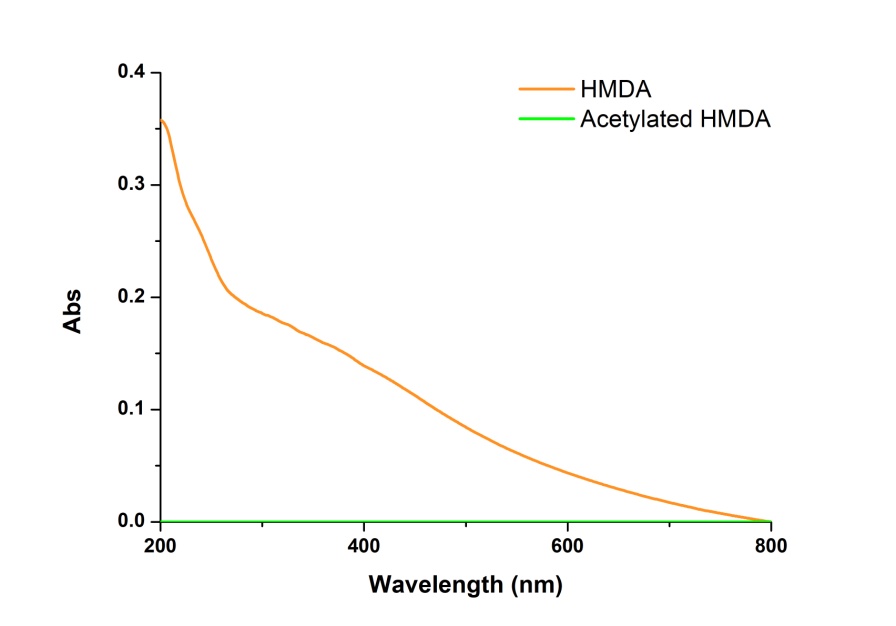


**Figure S1.** UV-vis absorption spectra of quartz substrates subjected to dip-coating for 24 h with 1 mM dopamine and HMDA (orange curve) or diacetyl derivative of HMDA (green trace) in carbonate buffer at pH=9.0.

**Figure S2.** Evolution of the UV-vis spectra of PDA/HMDA films in the presence of periodate in 0.05 M acetate buffer pH 5.0 or 0.05 M carbonate buffer pH 9.0 at a selected wavelength (400 nm).


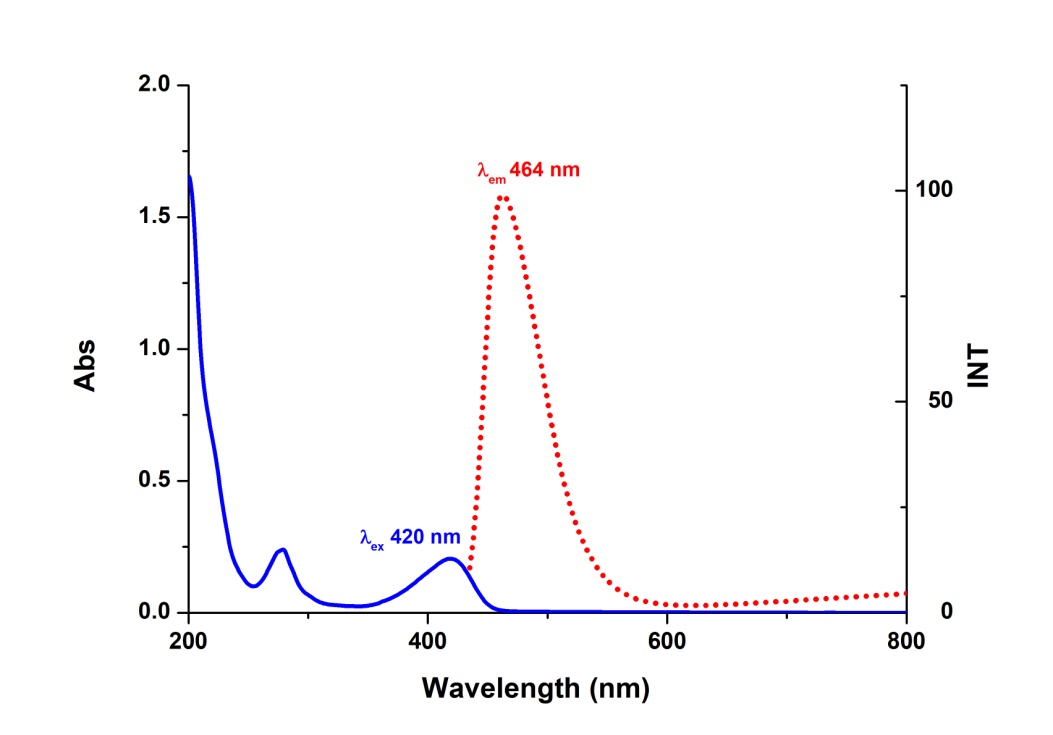


**Figure S3.** UV-vis (blue trace) and fluorescence spectra (red dot trace) (λ_ex_ = 420 nm) of the reaction mixture of DA/HMDA in the presence of resorcinol at 1 mM concentration taken 4 h under stirring in 50 mM carbonate buffer, pH= 9.0 (1:10 v/v dilution in water).

**Scheme S1.** Oxidative coupling of dopamine with resorcinol leading to fluorescent methanobenzofuroazocinone products.
